# High-temperature ferroelastic phase transition in a perovskite-like complex: [Et_4_N]_2_[PbBr_3_]_2_†

**DOI:** 10.1039/c9ra00804g

**Published:** 2019-04-02

**Authors:** Yuan Huang, Jie Yang, Zi-jian Li, Kun Qian, Feng Sao

**Affiliations:** College of Pharmacy, Jiangxi University of Traditional Chinese Medicine Nanchang 330004 P. R. China qk0876@hotmail.com; Shanghai Institute of Applied Physics, Chinese Academy of Sciences Shanghai 201800 P. R. China lizijian@sinap.ac.cn; Laboratory of Modern Preparation of TCM, Ministry of Education, Jiangxi University of Traditional Chinese Medicine Nanchang 330004 P. R. China shaofeng0729@163.com

## Abstract

A new lead-bromide hybrid organic–inorganic complex [Et_4_N]_2_[PbBr_3_]_2_ (Et = ethyl) was synthesized, and its crystal structures could be described as a distorted perovskite-like one (at room temperature phase and at high temperature phase) and a step-like dielectric anomaly was obtained at around 375 K upon heating and 367 K upon cooling. It underwent a reversible structural phase transition with the Aizu notation of 6/*mmmF*2/*m* belonging to one of the 94 species of ferroelastic phase transitions, displayed switchable dielectric behaviors triggered by the motion or reorientation of the tetraethylammonium cations and the displacement of Pb^2+^ and Br^−^ ions in a solid-state crystal. Ferroelastic domain walls were also observed in atomic-force microscopy. Differential scanning calorimetry, dielectric measurements and variable-temperature X-ray structure determinations indicated this complex exhibited a dielectric anomaly associated with the structural phase transition. All of these demonstrate its potential application as a temperature switchable molecular dielectric material in the ferroic-related field.

## Abstract 2

A new lead-bromide hybrid organic–inorganic complex [Et_4_N]_2_[PbBr_3_]_2_ (Et = ethyl) was synthesized, and its crystal structures could be described as a distorted perovskite-like one and a step-like dielectric anomaly was obtained at around 375/367 K.

## Graphical abstract

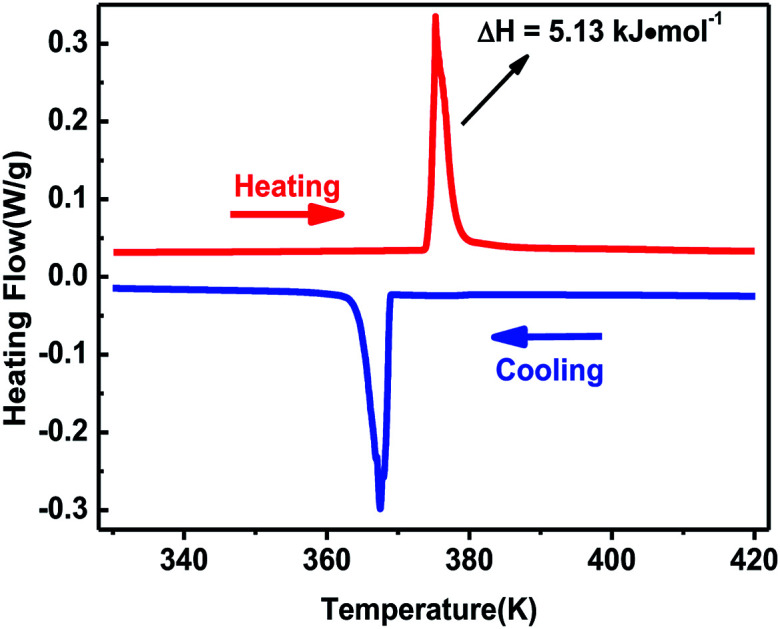

## Introduction

Ferroelastic materials have drawn special attention due to their prominent role in the construction and development of multiferroic materials with promising potential applications such as mechanical switches.^1–5^ Ferroelasticity is a phenomenon in which a material may exhibit a spontaneous strain. In ferroics, ferroelasticity is the mechanical equivalent of ferroelectricity and ferromagnetism. When stress is applied to a ferroelastic material, a phase change will occur in the material from one phase to an equally stable phase, either of different crystal structure (*e.g.* cubic to tetragonal), or of different orientation (a ‘twin’ phase). This stress-induced phase change results in a spontaneous strain in the material. The shape-memory effect and superelasticity are manifestations of ferroelasticity. Nitinol (nickel titanium), a common ferroelastic alloy, can display either super elasticity or the shape-memory effect at room temperature, depending on the nickel/titanium ratio.^6–34^ As it is fairly a difficult experimental task to measure the ferroelastic hysteresis with any acceptable degree of accuracy, it has become conventional to call a material ‘ferroelastic’ when a phase transition belonging to one of the 94 species of ferroelastic phase transitions defined by Aizu occurs (or may be thought to occur) which may convincingly generate ferroelasticity.^35^ Moreover, ferroelastic domain, which can be observed as a result of the reduction in symmetry between the paraelastic and ferroelastic phases, is an important ingredient in functionalities of ferroelastic materials.^36^

Many of these materials are oxides and some halide perovskites, such as lead zirconate titanate (PZT) and BaTiO_3_. In recent years, the organic–inorganic perovskite-type hybrids attract increasing attention owing to their diverse applications in data communication, rewritable optical data storage, thermal energy storage and mechanical energy transfer, *etc.*^7,37–48^ For example, an organic–inorganic perovskite piezoelectric, Me_3_NCH_2_ClMnCl_3_, exhibits a piezoelectric coefficient *d*_33_ of 185 pC N^−1^. Such a large *d*_33_ makes it a molecular material with a piezoelectric coefficient comparable with that of piezoelectric ceramics such as BaTiO_3_ and a high phase-transition temperature of 406 K beyond that of BaTiO_3_ (393 K).^37^ Another two-dimensional (2D) perovskite ferroelectric, (C_4_H_9_NH_3_)_2_CsPb_2_Br_7_, shows a high Curie temperature (*T*_C_ = 412 K) beyond that of BaTiO_3_ (393 K) and notable spontaneous polarization (4.2 μC cm^−2^).^38^ In addition, an organic–inorganic perovskite-type compound [(CH_3_)_3_PCH_2_OH][CdBr_3_] exhibits a ferroelastic phase transition at 339 K. The origin of the phase transition can be attributed to the motion or reorientation of the [(CH_3_)_3_PCH_2_OH]^+^ cations and the displacement of Cd^2+^ and Br^−^ ions in solid-state crystal.^7^ The two-dimensional multilayered perovskite ferroelectric, (C_4_H_9_NH_3_)_2_(CH_3_NH_3_)_2_Pb3Br_10_, shows prominent detecting behaviors including extremely low dark current (10^−12^ A), large on/off current ratio (2.5 × 10^3^) and highly-fast response rate (150 μs). These merits are superior to integrated detectors of other 2D perovskites, and compete with the most active CH_3_NH_3_PbI_3_.^44^

Encouraged by these perovskite-type compounds, we synthesized an organic–inorganic hybrid perovskite-like complex [Et_4_N]_2_[PbBr_3_]_2_ (1). Dielectric measurement and variable-temperature structural analyses indicated complex 1 showed a dielectric anomaly associated with the structural phase transition. Complex 1 underwent a reversible dielectric phase transition with the Aizu notation of 6/*mmmF*2/*m* belonging to one of the 94 species of ferroelastic phase transitions, displayed switchable dielectric behaviors triggered by the motion or reorientation of the tetraethylammonium cations and the displacement of Pb^2+^ and Br^−^ ions in solid-state crystal. Ferroelastic domain walls were also observed in atomic-force microscopy (AFM).

## Experimental

### Synthesis

All starting reagents and solvents employed for synthesis were commercially available and used without further purification. Complex 1 was synthesized as needle-shaped single crystals by slow evaporation of the mixture solution of lead bromide and tetraethylammonium bromide in the molar ratio of 1 : 1 in a 40% hydrobromic acid solution.

### IR spectrum and elemental analyses

The IR spectrum was recorded in the range of 4000–400 cm^−1^ on a Tensor 27 OPUS (Bruker) FT-IR spectrometer from KBr pellets. Elemental analyses (C, H, and N) were performed on a Model 240 Perkin-Elmer elemental analyzer.

### Thermal measurements

Thermogravimetric analysis was recorded on a DSC/DTA-TG SDT-Q600 instrument at the heating rate of 20 K min^−1^ under nitrogen atmosphere from 300 to 860 K in alumina crucibles. Differential scanning calorimetry (DSC) was performed by heating and cooling the polycrystalline samples on a Perkin-Elmer Diamond DSC instrument in the temperature range 330–420 K with a heating and cooling rate of 10 K min^−1^ under nitrogen at atmospheric pressure in aluminum crucibles.

### Single crystal structure determination

X-ray data for the title complex 1 was collected on a Bruker Smart APEX II diffractometer with MoK_α_ radiation (*λ* = 0.71073 Å) by using an φ–ω scan mode at 296 K and with CuK_α_ radiation (*λ* = 0.15406 Å) at 393 K. Data processing including empirical absorption corrections was performed using the Crystal Clear software package (Rigaku, 2005). Absorption corrections were applied using the SADABS program.^49^ The structures were solved by Direct Methods^50^ with the SHELXTL program and refined by full matrix least-squares techniques on *F*^2^ with the SHELXTL program.^50–52^ Non-H atoms were refined anisotropically using all reflections with *I* > 2*σ*(*I*). All H atoms were generated geometrically and refined using a “riding” model with *U*_iso_ = 1.2*U*_eq_ (C and N). Crystallographic data and structure refinement Details at 296 and 393 K are given in Table S1.†

### Dielectric measurements

The complex dielectric permittivity *ε* (*ε* = *ε*′ − i*ε*′′) was measured on a Tonghui TH2828A at frequencies of 500 to 1 MHz with an applied alternating electric field of 1 V. The powder-pressed pellets and single crystal sample deposited with silver conducting glue were used in dielectric measurements.

### AFM determination

To observe the possible boundary microstructure of complex 1 by atomic force microscopy,^53–62^ well-developed crystals with acceptable size were drawn from solution and the freshly cleaved crystal wafer was fixed on the AFM sample holder. All the images were collected using a Nanoscope, IIIa MultiMode AFM instrument of Digital Instruments Incorporation, with DI square pyramidal Si_3_N_4_ tips on V-shaped cantilevers of 100 mm in length with 0.06 N m^−1^ spring constants. Since the samples may result in scratching images after being scanned many times, all the zoomed areas were scanned only once to collect the possible intrinsic micro-structural images.

## Results and discussion

### IR properties

The structure of complex 1 was characterized by IR spectroscopy. The infrared spectrum of complex 1 was consistent with its formulation (Fig. S1†). Strong IR bands centred at 2986 cm^−1^ can be assigned to the *ν*_C–H_ stretching vibration of –CH_3_ groups. Strong IR bands centred at 1390 to 1482 cm^−1^ can be assigned to *ν*_C–H_ flexural vibration of the tetraethylammonium cations.

### Thermal properties

**Fig. 1:**
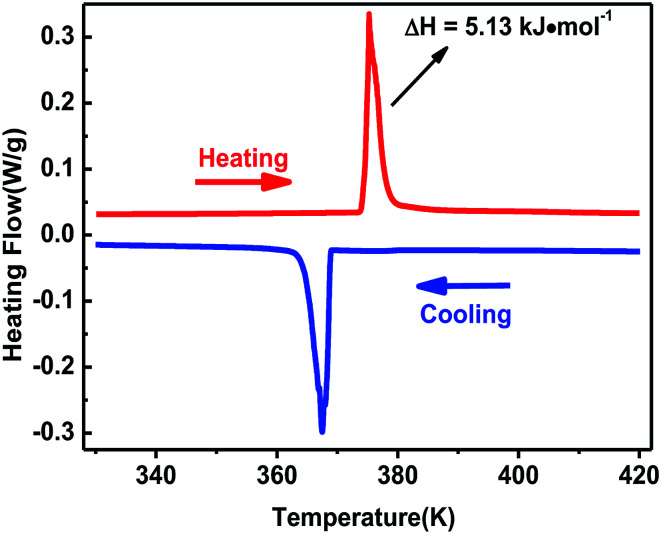
DSC curves of complex 1 shown in the temperature ranging from 330 to 420 K.

The curve of TG-DTA was tested at the temperature ranging from 300 to 860 K under nitrogen atmosphere. As shown in Fig. S2,† the weight curve shows that complex 1 began to melt till about 600 K, and the heat flow curve shows that a peak occured at 375 K which indicates a phase change may exist. The phase transition behavior of complex 1 was confirmed by differential scanning calorimetry in the temperature ranging from 330 to 420 K. A couple of heat anomalies at 375 K upon heating and 367 K upon cooling were observed in the DSC curves (Fig. 1), indicating that complex 1 underwent a reversible phase transition at around *T* = 375 K upon heating. For convenience, we labelled the phase above 375 K as the high-temperature phase (HTP), and the phase below 367 K as room-temperature phase (RTP).

On the basis of DSC curves, the average entropy change Δ*S* was estimated to be approximately 13.69 J mol^−1^ K^−1^. According to the Boltzmann equation Δ*S* = *R* ln(*N*), where *R* is the gas constant and *N* represents the proportion of numbers of possible orientations for the whole disordered system. The value *N* is calculated to be 5.19, suggesting the order-disorder phase transition feature of complex 1, which will be confirmed by the structural analysis.

### Single crystal structure determination

**Fig. 2:**
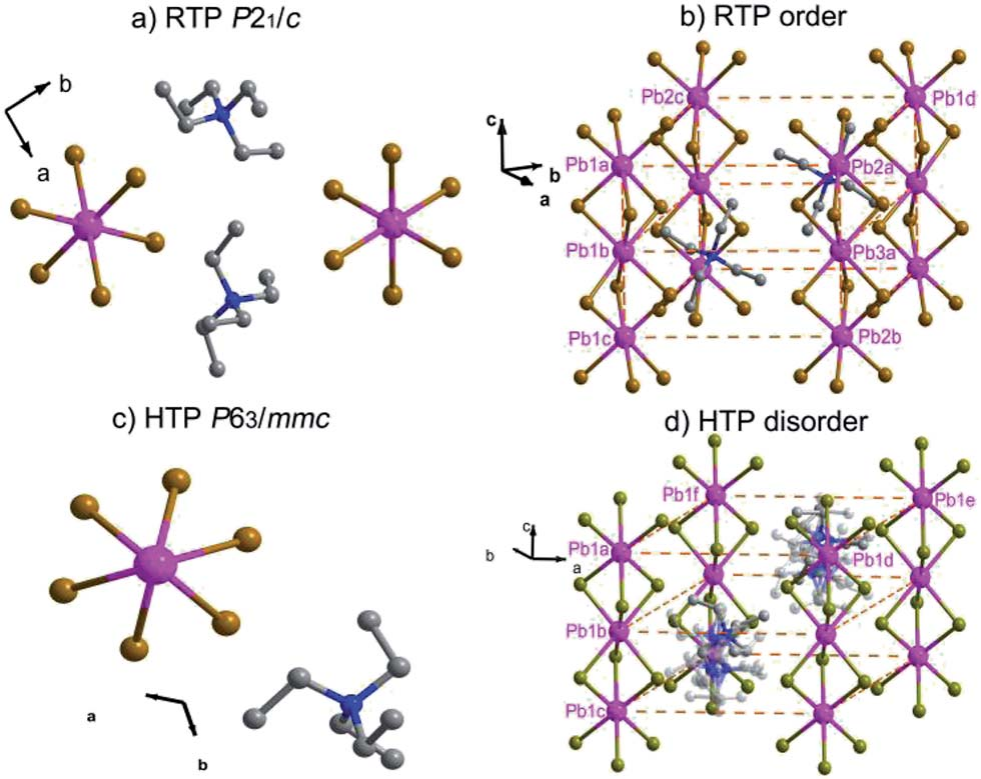
(a) Molecular structures of complex 1 (296 K). (b) Packing diagram of complex 1 (296 K). (c) Molecular structures of complex 1 (393 K). (d) Packing diagram of complex 1 (393 K) (the hydrogen atoms bonded to the C atoms omitted for clarity).

In order to reveal the details of structural phase transition, crystal structures of complex 1 were collected by variable-temperature X-ray single-crystal diffraction analysis at two different temperatures 296 K and 393 K, respectively. It reveals that complex 1 belongs to the monoclinic centrosymmetric space group *P*2_1_/*c* at 296 K and the hexagonal centrosymmetric space group *P*6_3_/*mmc* at 393 K (Table S1†). In both phases, each Pb atom is coordinated by six bridging Br^−^ ions to give one-dimensional chains running along *c* axis (Fig. 2a, Fig. 2c). From RTP to HTP with an increasing temperature, the crystal structures in both phases can be roughly described as distorted perovskite-like ones (Fig. 2b and d). Upon heating, all the Pb–Br bond lengths and Br–Pb–Br bond angles changed within the normal ranges. Especially, the tetraethylammonium cations show changes from RTP to HTP, from order to a six-fold disorder, giving rise to the higher symmetry.

In the RTP, each Pb atom, lying in the 2_1_ screw axis, is octahedrally coordinated by six bridging Br^−^ atoms with Pb–Br bond distances varying from 2.949(9) to 3.133(9) Å and Br–Pb–Br angles varying from 80.49(3) to 99.51(3)° (Table S2†). The distances of N and C atoms vary from 1.479(8) to 1.541(8) Å, the angles of C–N–C vary from 106.20(5) to 112.20(5)°. Whereas in the HTP, with each Pb1 atom occupying the 3*m* symmetry site, the Pb–Br bond distance changes to 3.030(1) Å and the Br–Pb–Br bond angles are in the range from 81.59(10) to 98.41(10)°. The C–N bond lengths vary from 1.480(2) to 1.500(2) Å and the C–N–C bond angles vary from 108.40(18) to 110.60(18)° (Table S3†). Whereas in the HTP, the cationic part is completely disordered, the tetraethylammonium cations exhibits disorder over six positions (Fig. 2d).

As shown in Fig. S3,† the stacking structure changes slightly due to movement of molecules. The Pb1a⋯Pb1c distance in the RTP increases little from 7.838 to 7.954 Å (Pb1a⋯Pb1c distance in the HTP). With the C, H, N atoms omitted for clarity in Fig. S3,† the neighbouring Pb1a⋯Pb2a distance (10.490 Å) in the RTP is obviously shorter than the corresponding Pb1a⋯Pb1d distance (10.595 Å) in the HTP, while the neighbouring Pb1a⋯Pb2c distance (10.545 Å) in the RTP is shorter than Pb1a⋯Pb1f distance (10.595 Å) in the HTP. Meanwhile, the Pb2c⋯Pb1a⋯Pb2a angle in the RTP changes from 62.37 to 60.00° (Pb1d⋯Pb1a⋯Pb1f angle in the HTP).

**Fig. 3:**
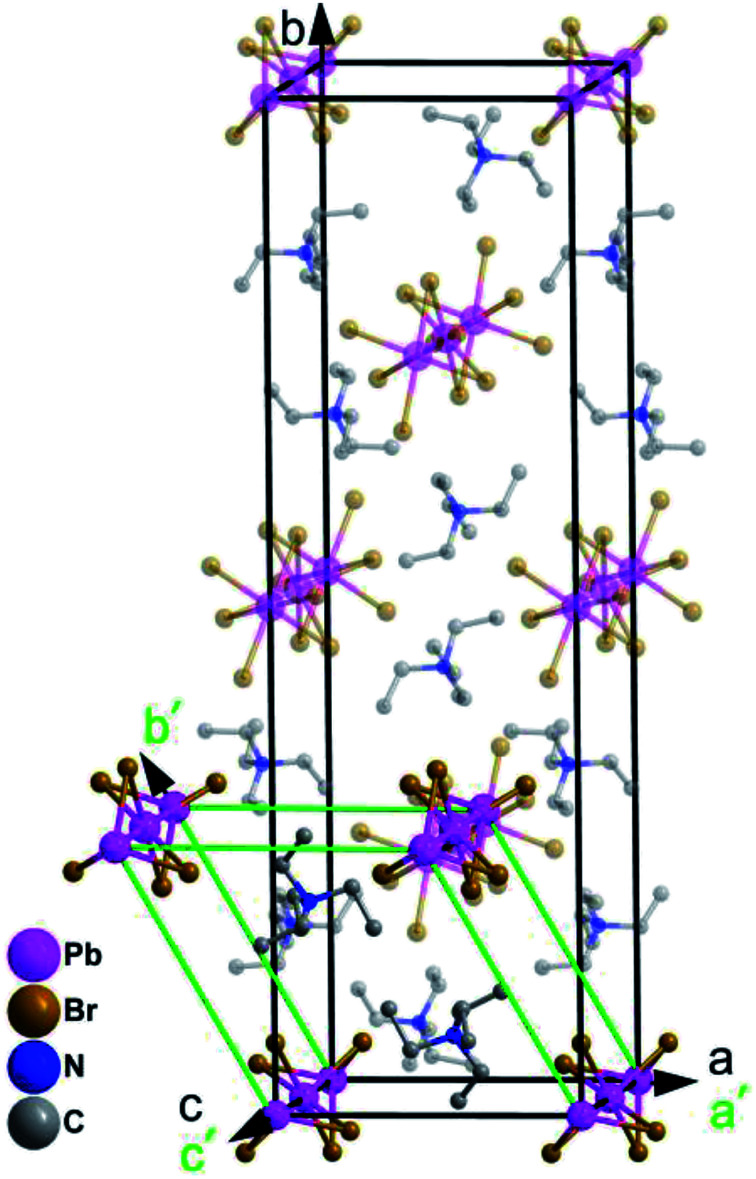
The room temperature crystal cell of complex 1 (outlined in black solid lines) and its high temperature unit cell (outlined in green solid lines).

The cage units per cell decrease from four (in the RTP) to two (in the HTP) during the heating process. What is noteworthy is that the structure parameters change tremendously from RTP to HTP [*a*, *b*, *c* (Å): 10.892(3), 35.883(10), 7.838(2) *vs.* 10.595(6), 10.595(6), 7.954(6)]. The comparison of the two phases is clearly shown in Fig. 3. The phase transition may be ascribed to the motion or rotation of tetraethylammonium cations and the displacement of Pb^2+^ and Br^−^ ions upon heating.

### Dielectric properties of complex 1

**Fig. 4:**
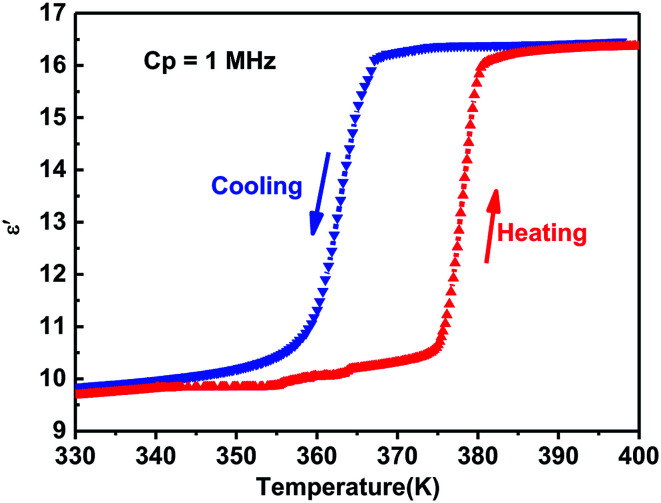
The dielectric constant (*ε*′) of the polycrystalline sample of complex 1 measured at 1 MHz.

Generally, phase transitions are always accompanied by anomalies of physical properties as responses to external stimuli. Hence, the temperature-dependent dielectric permittivity measurements of complex 1 were performed on polycrystalline at different frequencies. The temperature dependence of the real part *ε*′ of the complex constant (*ε* = *ε*′ − i*ε*′′, *ε*′′ is the imaginary part) was measured with the temperature ranging from 330 to 400 K shown in Fig. 4. For complex 1, obvious step-like dielectric anomalies at around 375 K upon the heating process and 367 K upon the cooling process are obtained. At 1 MHz, upon the heating process the *ε*′ kept stable with a value of about 10.0 when the temperature was ranging from 330 to 375 K, which corresponds to a low dielectric state. At 375 K, the *ε*′ value rapidly increases to reach a value of about 16.1 and transfers to a high dielectric state. Upon the cooling process, the *ε*′ kept stable with a value of about 16.1 when the temperature was ranging from 400 to 367 K, which corresponds to a high dielectric state. At 367 K, the *ε*′ value rapidly decreases to reach a value of about 10.0 and transfers to a low dielectric state. The value of about 16.1 in the high dielectric state that is almost 1.6 times that obtained in the low dielectric state. The dielectric anomalies are in conformity with the thermal anomalies of DSC measurement (Fig. 1).

**Fig. 5:**
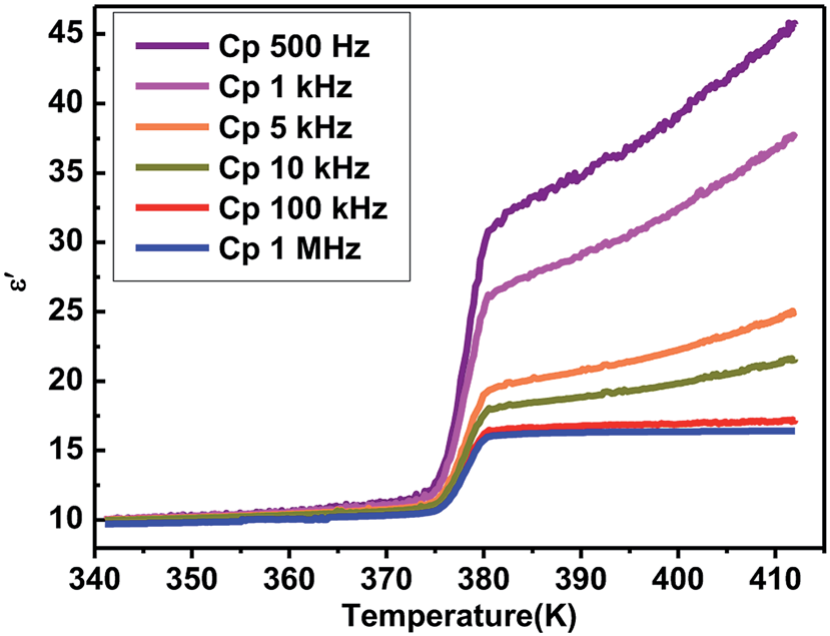
The temperature-dependence of the dielectric constant (*ε*′) of the polycrystalline sample of complex 1 measured at the temperature ranging from 340 to 410 K upon the heating process at 500 Hz to 1 MHz.

Furthermore, the dielectric constant (*ε*′) of the complex dielectric permittivity of the polycrystalline sample measured at 500, 1 K, 10 K, 100 K, 500 K, 1 MHz at the temperature ranging from 340 to 410 K is shown in Fig. 5. The variable-frequency dielectric response shows a frequency-dependent phenomenon. At different frequencies, the *ε*′ keeps a little increasing with the value of about 10.0 below 375 K, which corresponds to a low dielectric state. When the temperature increases to 375 K, a striking step-like anomaly appears, which corresponds to a high dielectric state. As the temperature increases further, *ε*′ value goes up a small increase. At the high dielectric state, the *ε*′ and *ε*′′ value decrease with the frequencies increase at the same temperature (Fig. 5 and S4†).

### Ferroelastic domain walls observed in AFM

**Fig. 6:**
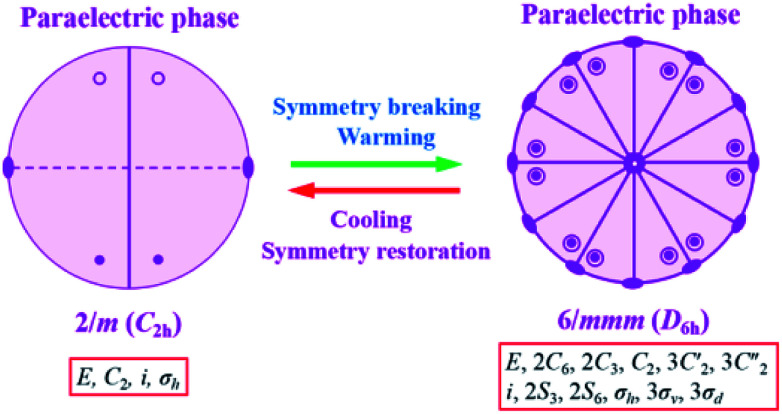
Spatial symmetry operation changes of complex 1 from the RTP (*P*2_1_/*c*) to the HTP (*P*6_3_/*mmc*).

It is noted that the unit cell parameters vary greatly from HTP to LTP, and the crystal system changes from hexagonal type to monoclinic type during the cooling process and space group change from *P*6_3_/*mmc* to *P*2_1_/*c.* The twenty four symmetry elements (*E*, 2*C*_6_, 2*C*_3_, *C*_2_, 3*C*′_2_, 3*C*_2_′′, *i*, 2*S*_3_, 2*S*_6_, *σ*_h_, 3*σ*_v_, 3*σ*_d_) in the HTP decrease to four (*E*, *C*_2_, *i*, *σ*_h_) in the RTP. According to Aizu notation, it can be classified into the 6/*mmmF*2/*m* species, and the phase transition should be ferroelastic according to the 94 species of ferroelastic phase transitions. The possible domain number is *q* = 24/4, hence the number of equivalent unique ferroelastic directions is up to 6. That is, the spatial orientations of six ferroelastic domains are considered as a result of symmetry reduction. The Curie symmetry principle tells us that the space group at the ferroelastic phase should be the subspace group at the paraelastic phase, *i.e.* its maximal non-isomorphic subgroups containing 1̄, 6/*m*, 2/*m*(3), 2/*m*(3), respectively. Furthermore, the number of spatial symmetry operations decrease from 24 to 4 during the symmetry breaking process (Fig. 6), in good agreement with macroscopic symmetry breaking.

**Fig. 7:**
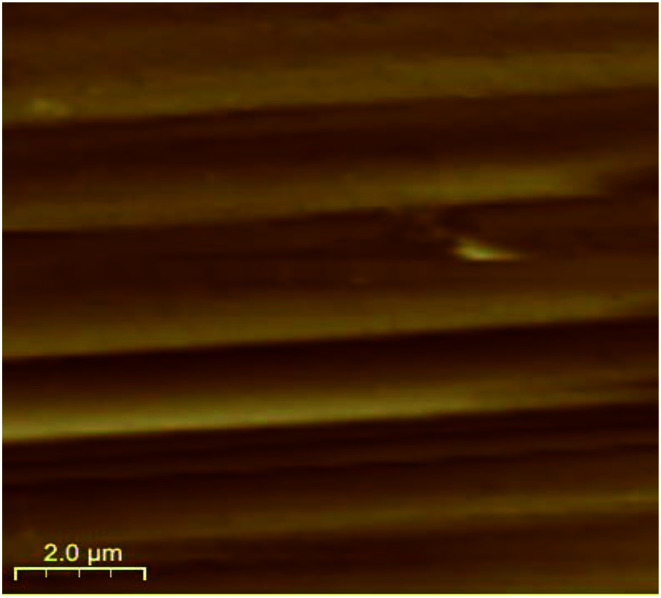
AFM image of a cleavage surface pattern in complex 1.

As a promising tool to observe the surface morphology down to the atomic dimensional scale, AFM has been employed to investigate the permissible domains and surface patterns correlated with the boundary structures. It is expected that the surface patterns may reflect the orientation of the domain structure. Here, a clear boundary-contrast AFM topography imaging was recorded in freshly crystal samples of complex 1. As shown in Fig. 7, AFM image of a cleavage surface pattern in complex 1 with the scanning area of 10.0 × 10.0 μm^2^, and one can observe the ferroelastic domain walls clearly. These ferroelastic domain walls are nearly parallel each other and distinctly reflect the orientation of the domain structure. The boundaries between two different states are well-defined, and the domains of the width of approximately 1.3 μm.

From the perspective of symmetry change, the transition fulfills the reverse group–subgroup relation. One analogous compound [(CH_3_)_3_PCH_2_OH][CdBr_3_] exhibits a ferroelastic phase transition at 339 K. Its symmetry change occurred with an Aizu notation of 6/*mmmFmmm*. Domain structures were observed and analysed.^7^ A similar behavior in (TMA)(TEA)MnBr_4_ suggests that ferroelastic phase exists in this crystal, and the ferroelastic domain structure consistent with Aizu species 
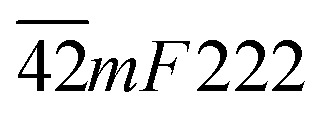
 was revealed.^11,14^ In another analogous compound [N(CH_3_)_4_][Cd(N_3_)_3_], its phase transition should be ferroelastic with an Aizu notation of *m*3*mF*2/*m*, and the number of equivalent unique ferroelastic directions is up to 12. There was no domain structure reported.^21^ An organic co-crystal with a perovskite-like architecture, dabco *p*-nitrophenol, undergoes a reversible ferroelastic phase transition with the Aizu notation of 2/*mF*1̄ at around 128 K. Clear-cut regions with different growth directions of units are presented by AFM, in which polydirectional growth might give rise to the permissible boundary or even domain walls in adjacent areas.^23^ The ferroelectrics NaKC_4_H_4_O_6_·4H_2_O (the crystal commonly known as Rochelle salt) exhibits two Curie points. The ferroelectric phase of this material is also ferroelastic and it exists between 255 and 297 K.^63^ Both the spontaneous polarization and strain rise with increasing temperature above the lower Curie point.^64^

## Conclusions

In summary, the present work has successfully demonstrated a new lead-bromide organic–inorganic hybrid complex [Et_4_N]_2_[PbBr_3_]_2_. The crystal structure of title complex can be described as a distorted perovskite-like one and exhibits a dielectric phase transition around 375 K upon heating and 367 K upon cooling. The results indicate that the dielectric phase transition is related to the motion or reorientation of the tetraethylammonium cations and the displacement of Pb^2+^ and Br^−^ ions in solid-state crystal under the stimuli of variable temperature. It reveals a reversible ferroelastic phase transition with the Aizu notation of 6/*mmmF*2/*m*. Ferroelastic domain walls were also observed in atomic-force microscopy. This compound can be regarded as an excellent temperature stimuli-responsive dielectric material with ferroelastic phase transition and affords a promising strategy to search new materials.

## Conflicts of interest

There are no conflicts to declare.

## Supplementary Material

RA-009-C9RA00804G-s001

RA-009-C9RA00804G-s002
