# Supplementary material for: High-temperature ferroelastic phase transition in a perovskite-like complex: [Et4N]2[PbBr3]2
Source: RSC Adv. 2019 Apr 2;9(18):10364–70. doi: 10.1039/c9ra00804g (PMC9062389; doi:10.1039/c9ra00804g)
Supplement: RA-009-C9RA00804G-s001 [file RA-009-C9RA00804G-s001.pdf]

Electronic Supplementary Material (ESI) for RSC Advances.
This journal is © The Royal Society of Chemistry 2019


# High-temperature ferroelastic phase transition in a perovskite-like complex: $[Et_4N]_2[PbBr_3]_2$

Yuan Huang, [a] Jie Yang, [a] Zi-jian Li, [b] Kun Qian, *[a] and Feng Sao *[c]

*a*. College of Pharmacy, Jiangxi University of Traditional Chinese Medicine, Nanchang, 330004, P. R. China, e-mail: qk0876@hotmail.com

*b*. School of Chemistry and Chemical Engineering, Shanghai Jiao Tong University, Shanghai, 200240, P. R. China, e-mail: lizjian@sjtu.edu.cn

*c*. Laboratory of Modern Preparation of TCM, Ministry of Education, Jiangxi University of Traditional Chinese Medicine, Nanchang, 330004, P. R. China, e-mail: shaofeng0729@163.com

**Synthesis of the title compound**

Tetraethylammonium bromide (1 mmol, 0.210 g) was dissolved in 4 mL 40% hydrobromic acid solution and lead bromide (1 mmol, 0.367 g) dissolved in 2 mL 40% hydrobromic acid solution, mixed the above solution and stirred for about twenty minutes. Filtered the mixture solution and kept at room temperature, white needle-shaped crystals were obtained by slow evaporation after one day.

**Figure Captions**

**Fig. S1** Infrared spectrum of solid complex **1** in KBr pellets recorded at room temperature.

**Fig. S2** Thermo-gravimetric and differential thermal analysis (TG-DTA) curves of complex **1** at the temperature ranging from 300 to 860 K**.**

**Fig. S3** Packing diagram of complex **1** at 296 K and 393 K. (a) The room temperature phase (296 K).(b) The high temperature phase (393 K). (all hydrogen atoms omitted for clarity)

**Fig. S4** The imaginary part of the dielectric constant (ε″) of complex **1** measured at the temperature ranging from 350 to 415 K upon heating at selected frequencies (500 Hz - 1 MHz).

This journal is © The Royal Society of Chemistry 2019

Please do not adjust margins



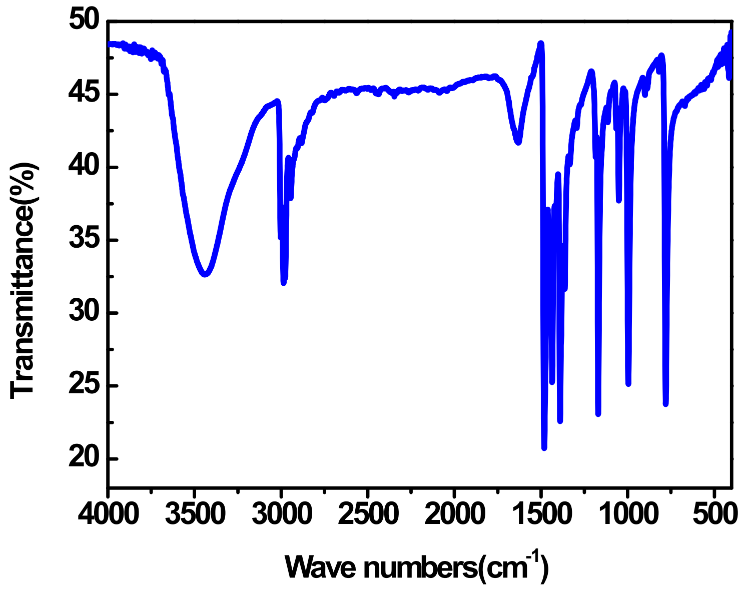


**Figure S1**

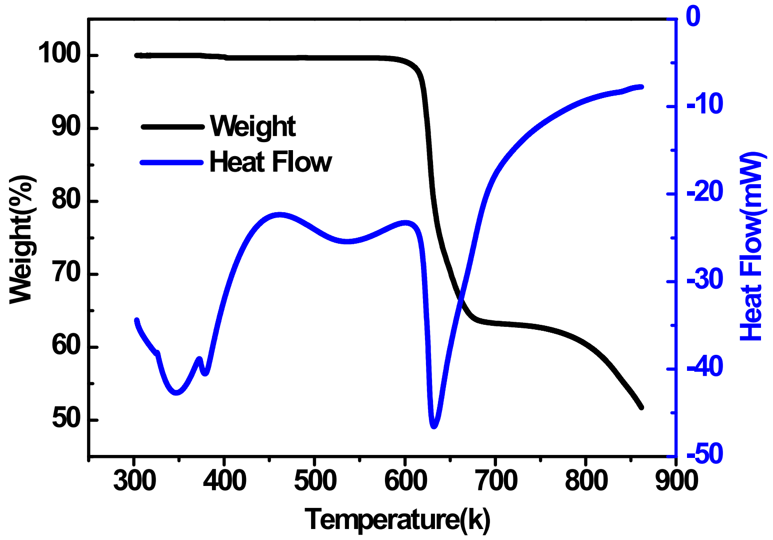


**Figure S2**



Please do not adjust margins

Please do not adjust margins



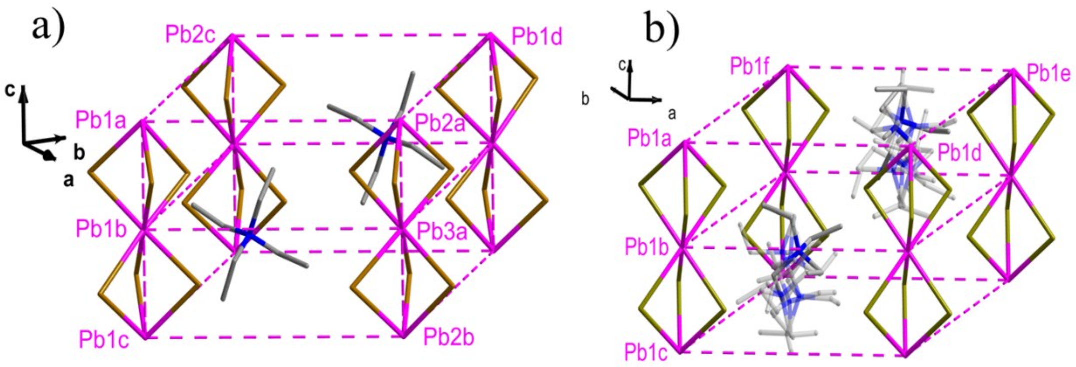


**Figure S3**

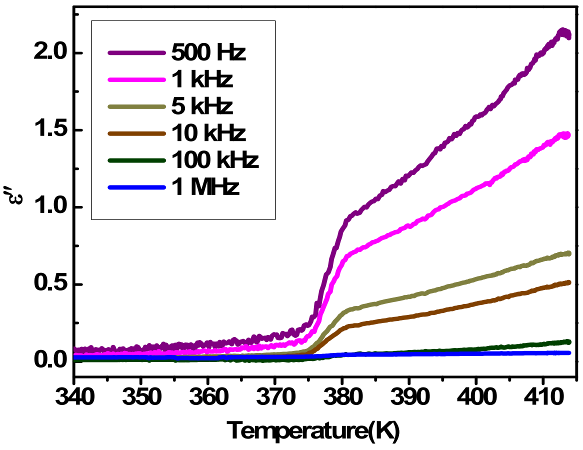


**Figure S4**

This journal is © The Royal Society of Chemistry 2019



Please do not adjust margins

Please do not adjust margins



**Table S1**. Summary of crystallographic data for complex **1**

| Empirical formula | $Pb_2Br_6C_{16}N_2H_{40}$ | $PbBr_3C_8NH_{20}$ |
|---|---|---|
| Formula weight | 1152.32 | 577.17 |
| T, K | 296(2) K | 393(2)K |
| Crystal system, space group | monoclinic, $P2_1/c$ | hexagonal, $P6_3/mmc$ |
| *a* , Å | 10.892(3) | 10.595(6) |
| *b*, Å | 35.883(10) | 10.595(6) |
| *c*, Å | 7.838(2) | 7.954(6) |
| *β*, deg | 100.7(3) | 90.0 |
| *V*, $Å^{-3}$ | 3010.1(14) | 773.3(11) |
| *Z* | 4 | 2 |
| $D_{calc.}$, mg $m^{-3}$ | 2.547 | 2.479 |
| $\mu$, $mm^{-1}$ | 19.142 | 30.030 |
| *F* (000), e | 2096 | 524 |
| *ϑ* range, deg | 1.13→25.00 | 4.82→72.04 |
| *hkl* range | -12→12 | -8→12 |
| | -42→42 | -12→12 |
| | -9→9 | -9→5 |
| Data/Restraints/parameters | 5305/0/246 | 316/129/89 |
| Reflections collected/unique | 20649/5305 | 2149/316 |
| $R_{int}$ | 0.0762 | 0.0948 |
| Goodness-of-fit on $F^2$ | 1.109 | 1.089 |
| $R_1/wR_2$ [$I > 2\sigma(I)$] | 0.0640/0.1499 | 0.0684/0.1252 |
| $R_1/wR_2$ (all data) | 0.1002/0.1612 | 0.0826/0.1358 |
| Largest peak and hole (e $Å^{-3}$) | 2.889/-6.167 | 1.589/-0.785 |

This journal is © The Royal Society of Chemistry 2019



Please do not adjust margins

Please do not adjust margins



**Table S2**. Selected bond lengths and angles of complex **1** at 296 K

| Bond lengths | Å | Bond angles | deg |
|---|---|---|---|
| Pb(1)-Br(1) | 2.976(9) | Br(4)$^{\#3}$-Pb(3)-Br(6) | 84.09(3) |
| Pb(1)-Br(2) | 2.949(9) | Br(4)-Pb(2)-Br(6) | 95.67(3) |
| Pb(1)-Br(3) | 3.012(10) | Br(4)-Pb(2)-Br(5) | 96.04(2) |
| Pb(2)-Br(4) | 2.996(9) | Br(4)$^{\#3}$-Pb(2)-Br(5) | 83.96(2) |
| Pb(2)-Br(5) | 3.065(9) | Br(4)-Pb(2)-Br(6)$^{\#3}$ | 84.33(3) |
| Pb(2)-Br(6) | 3.065(9) | Br(5)$^{\#3}$-Pb(2)-Br(6) | 99.51(3) |
| Br(2)-Pb(1)$^{\#1}$ | 3.133(9) | Br(5)-Pb(3)-Br(6) | 80.63(3) |
| N(1)-C(1) | 1.541(8) | Br(2)-Pb(1)-Br(1) | 95.17(3) |
| N(1)-C(3) | 1.533(7) | Br(1)-Pb(1)-Br(3) | 83.23(3) |
| N(1)-C(5) | 1.492(7) | Br(2)-Pb(1)-Br(3) | 94.52(3) |
| N(1)-C(7) | 1.518(8) | Br(5)-Pb(2)-Br(6) | 80.49(3) |
| N(2)-C(9) | 1.479(8) | Br(3)-Pb(1)-Br(2)$^{\#2}$ | 82.05(3) |
| N(2)-C(11) | 1.505(8) | C(9)-N(2)-C(13) | 106.2(5) |
| N(2)-C(13) | 1.490(9) | C(13)-N(2)-C(11) | 112.2(5) |
| N(2)-C(15) | 1.497(9) | | |

Symmetry transformations used to generate equivalent atoms: $^{\#1}$ *x*, -*y* + 1/2, *z* + 1/2; $^{\#2}$ *x*, -*y* + 1/2, *z* - 1/2; $^{\#3}$ -*x*, -*y*, -*z* + 1.



Please do not adjust margins

Please do not adjust margins



**Table S3.** Selected bond lengths and angles of complex **1** at 393 K

| Bond lengths | Å | Bong angles | deg |
|---|---|---|---|
| Pb(1)-Br(1) | 3.030(4) | Br(1)$^{\#1}$-Pb(1)-Br(1)$^{\#2}$ | 81.59(10) |
| Pb(1)-Br(1)$^{\#2}$ | 3.030(4) | Br(1)$^{\#2}$-Pb(1)-Br(1)$^{\#3}$ | 98.41(10) |
| Pb(1)-Br(1)$^{\#3}$ | 3.030(4) | C(1) -N(1)-C(3) | 110.6(18) |
| N(1)-C(1) | 1.48(2) | C(1) -N(1)-C(5) | 109.5(18) |
| N(1)-C(3) | 1.49(2) | C(5)-N(1)-C(7) | 108.4(18) |
| N(1)-C(5) | 1.50(2) | C(3)-C(4) | 1.551(12) |
| N(1)-C(7) | 1.50(2) | C(5)-C(6) | 1.550(12) |
| C(1)-C(2) | 1.549(12) | C(7)-C(8) | 1.549(12) |

Symmetry transformations used to generate equivalent atoms: $^{\#1}$ *y*, -*x* + *y*, -*z* + 3; $^{\#2}$ -*y*, *x* - *y*, *z*; $^{\#3}$ -*x* + *y*, -*x*, *z*.



Please do not adjust margins
